# Testing the Effectiveness of a Primary Care Intervention to Improve Uptake of Colorectal Cancer Screening: A Randomized Controlled Trial Protocol

**DOI:** 10.2196/resprot.7432

**Published:** 2017-05-10

**Authors:** Natalie Dodd, Mariko Leanne Carey, Elise Mansfield, Christopher Oldmeadow

**Affiliations:** ^1^ University of Newcastle School of Medicine and Public Health Faculty of Health and Medicine Callaghan Australia; ^2^ Hunter Medical Research Institute New Lambton Heights Australia; ^3^ Hunter Medical Research Institute Clinical Research Design, Information Technology and Statistical Support New Lambton Heights Australia

**Keywords:** clinical trial, colorectal cancer, early detection of cancer, general practice, primary care, primary care provider

## Abstract

**Background:**

Screening for colorectal cancer (CRC) significantly reduces mortality associated with this disease. In Australia, the National Bowel Cancer Screening Program provides regular fecal occult blood tests (FOBT) for those aged 50 to 74 years, however, participation rates in the program have plateaued at 36%. Given low uptake in the National Bowel Cancer Screening Program, it is necessary to explore alternate methods to increase CRC screening rates. Primary care is a promising adjunct setting to test methods to increase CRC screening participation. Primary care guidelines support the recommendation and provision of CRC screening to primary care patients. Those in the National Bowel Cancer Screening Program target age range frequently present to their primary care provider.

**Objective:**

This study tests the effect that a multicomponent primary care–based intervention has on CRC screening uptake when compared to usual care.

**Methods:**

Primary care patients presenting for an appointment with their primary care provider complete a touchscreen survey to determine eligibility for the trial. Those aged 50 to 74 years, at average risk of CRC, with no history of CRC or inflammatory bowel disease, who have not had an FOBT in the past 2 years or a colonoscopy in the past 5 years are eligible to participate in the trial. Trial participants are randomized to the intervention or usual care group by day of attendance at the practice. The intervention consists of provision of an FOBT, printed information sheet, and primary care provider endorsement to complete the FOBT. The usual care group receives no additional care.

**Results:**

The primary outcome is completion of CRC screening 6 weeks after recruitment. The proportion of patients completing CRC screening will be compared between trial groups using a logistic regression model.

**Conclusions:**

CRC screening rates in Australia are suboptimal and interventions to increase screening participation are urgently required. This protocol describes the process of implementing a multicomponent intervention designed to increase CRC screening uptake in a primary care setting.

**Trial Registration:**

Australian New Zealand Clinical Trials Registry ACTRN12616001299493; https://anzctr.org.au/Trial/Registration/TrialReview.aspx?id=371136&isReview=true (Archived by WebCite at http://www.webcitation.org/6pL0VYIj6). Universal Trial Number U1111-1185-6120.

## Introduction

Globally, colorectal cancer (CRC) is the third most common cancer in men and the second most common cancer in women [1]. Overall, it is the fourth leading cause of cancer death [2]. Worldwide, 1.4 million people are diagnosed with CRC every year, and 694,000 die as a result [2]. In Australia, CRC is the second most diagnosed and second most common cause of cancer death [3]. In 2012, 14,958 Australians were diagnosed with CRC and in 2013, 4162 died as a result of CRC [3].

The effectiveness of CRC screening using a fecal occult blood test (FOBT) has been established in several large randomized controlled trials (RCTs) [4-7]. Biennial FOBT screening reduces mortality from CRC by 13% to 33% [4-8]. FOBT is an affordable, accessible form of screening that can be completed by an individual in the privacy of their own home. Studies in the United States [9] and Israel [10] have found that the majority of participants prefer FOBT compared to other screening methods such as colonoscopy. Participants report that they prefer FOBT because it is convenient, affordable, less time-consuming, less painful when compared to other screening modalities, and requires no bowel preparation [9-11]. In Australia, guidelines recommend biennial FOBT for people aged 50 years and above who are at average risk of CRC [12].

Given the benefits associated with CRC screening, many countries, including Australia, have implemented population-based screening programs [13]. Population-based screening programs can be defined as those that provide a simple test to detect early signs of disease to all individuals in a target group, usually defined by age [14]. In Australia, those aged 50 to 74 years are mailed an invitation and FOBT kit as part of the federally managed National Bowel Cancer Screening Program [13]. Briefly, the program mails individuals an immunochemical FOBT, instructions, and a reply paid envelope. Completed tests are sent to a central processing laboratory. A reminder letter is sent to those not returning a test within 8 weeks [13]. Invitees returning a completed FOBT are able to nominate their primary care provider to receive test results.

The impact of this and other population-based screening programs is dependent upon achieving high rates of initial uptake and repeat screening among invitees. However, the most recent National Bowel Cancer Screening Program monitoring report indicated that, of the 1.4 million people sent an FOBT in 2013-2014, only 36% returned a completed FOBT [13]. Given this, there is an urgent need to explore ways to improve engagement in CRC screening.

Primary care is a potential setting to increase CRC screening participation. Primary care providers have frequent contact with those in the target age group for CRC screening [15], and giving advice on preventive care is perceived by patients as a key part of the primary care provider’s role [16]. Primary care guidelines [17-19] recommend that providers play a role in promoting CRC screening by assessing risk based on family history and providing screening advice and test referral. Despite this, a large proportion of primary care patients in Australia have not been screened at the recommended interval [20]. This suggests that in Australia, as in other countries, CRC screening advice is not routinely delivered in the primary care setting [21-23]. This may be due to a range of factors including limited time within the consultation [24-26], perceived lack of patient interest in conversations about CRC screening [21], and cultural barriers [27].

Systematic reviews have identified strategies that are effective for increasing CRC screening uptake in the primary care setting [28-31]. Two reviews concluded that supplying patients with free FOBT when they attended an appointment with their physician resulted in an increase in CRC screening uptake by 15% to 42% when compared to usual care [29,30]. Further, RCTs that included paper-based information on CRC screening using an FOBT also significantly increased CRC screening in the intervention group when compared to those that included no paper-based information or usual care [32,33]. RCTs have found that primary care provider endorsement (ie, recommendation to take part in screening) as part of an organized screening program invitation is associated with increased CRC screening uptake when compared to standard invitations [34,35]. Most studies, however, have evaluated primary care provider endorsement in the context of mail-based interventions [31,36]. Face-to-face endorsement within the context of a primary care consultation may have greater impact on screening uptake. While reviews have identified a number of potentially effective primary care–based strategies for increasing CRC screening, the majority of studies using opportunistic strategies have taken place in the United States [30,36]. Given that the United States has a different health care system than Australia, it is unclear how generalizable these findings are to the Australian primary care setting.

Building upon current evidence, this study incorporates effective strategies to deliver a multicomponent intervention to increase CRC screening in the Australian primary care setting. The intervention comprises a novel combination of printed information on screening, the provision of a free point-of-care FOBT, and face-to-face primary care provider endorsement of screening.

## Methods

### Hypotheses

Our first hypothesis is that compared to usual care participants, those allocated to the intervention group will report a 20% higher rate of CRC screening uptake at 6-week follow-up. Our second hypothesis is that compared to usual care participants, those in the intervention group will show a greater increase in knowledge from baseline to follow-up.

**Figure 1:**
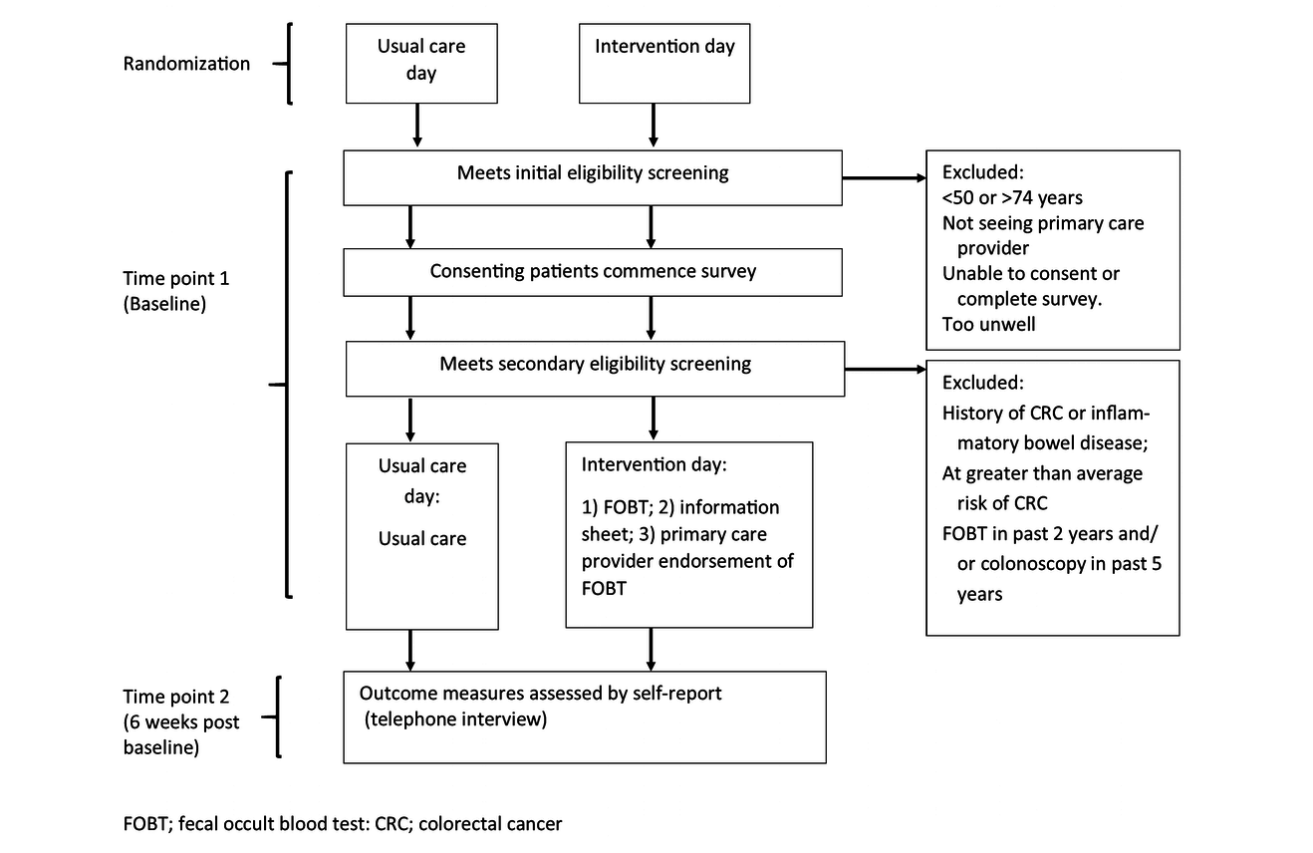
Flow of participants.

### Trial Design and Setting

This study is taking place in 5 primary care practices in New South Wales, Australia. A cluster RCT design is being used with consenting participants allocated to the intervention or usual care group depending on the day they attend the practice (see Figure 1).

### Practice Eligibility and Recruitment

A convenience sample of primary care clinics has been recruited for this study. To ensure adequate throughput of patients, eligible practices were required to have at least 2 full-time equivalent primary care providers. Primary care practice managers were sent an invitation and information statement via email. Nonresponding practices were followed up by telephone. Of 18 invited practices, 5 agreed to participate. Practice managers and primary care providers within each practice received an information statement and provided written informed consent.

### Randomization

Using a computer-generated randomization table with block sizes of 4, recruitment days are randomly allocated using a 1:1 ratio to intervention or usual care. Randomization by day rather than individual participant was selected to minimize potential for contamination between experimental groups. The allocation cannot be concealed from the research assistant conducting participant recruitment; however, these staff do not have access to the assignment schedule and are only made aware of allocation the day prior to attending the practice.

### Participant Eligibility Criteria

Those who (1) are aged 50 to 74 years, (2) have no personal history of bowel cancer or inflammatory bowel disease, (3) are at average risk of CRC, and (4) have not had an FOBT in the past 2 years or a colonoscopy in the past 5 years are eligible to participate in the trial.

### Exclusion Criteria

Those who are (1) not seeing a primary care provider, (2) too unwell, (3) unable to complete the touchscreen survey, or (4) unable to speak and read English sufficiently are excluded from the trial.

### Training of Staff

All training is delivered by one of the chief investigators prior to any recruitment. A training manual for research assistants developed by the research team is used for both training and as a reference during recruitment and follow-up. All research assistants receive face-to-face and on-site training in recruitment and data collection procedures. Reception staff are provided with an overview of the project as well as the process to identify eligible patients and how to refer them to the research assistant. A sign reminding reception staff to check patients for eligibility is placed at their workstation. One of the chief investigators attends a regular staff meeting at each practice to brief the primary care providers about the project and provide them with a dialogue sheet to encourage FOBT completion by patients assigned to the intervention group.

### Procedure for Assessing Eligibility

A two-stage process determines trial eligibility. Initial patient eligibility screening begins when reception staff flag patients in the target age range to the research assistant, who invites patients in the waiting room to complete a touchscreen survey to assess trial eligibility, and if eligible, to take part in the trial. Patients are provided with an information statement and allowed time to ask any questions they may have about the trial. Those providing written consent complete a 10-minute touchscreen survey in the waiting room prior to their primary care appointment. Assistance to complete the touchscreen survey is provided by the research assistance as required. Study participants do not receive compensation for their time in the study.

Second-stage patient eligibility screening is performed during the touchscreen computer survey:

1. No personal history of bowel disease: Participants are asked whether they have ever received a diagnosis of bowel cancer or inflammatory bowel disease (yes/no). FOBT screening recommendations related to biennial FOBT are only relevant to asymptomatic individuals with no prior history of CRC. Therefore, those who respond “yes” are excluded.
2. Average risk for CRC: Participants are asked “How many of your first-degree relatives have ever been diagnosed with bowel cancer?” (0, 1, 2 or more) and “Were any of your relatives who have had bowel cancer diagnosed before the age of 55?” (yes/no). Based on criteria in the Australian National Health and Medical Research Council guidelines [12], those who report no relatives diagnosed with CRC aged younger than 55 years and up to one first-degree relative diagnosed with CRC at any age are classified as average risk for CRC. Those classified as at higher risk of CRC are excluded, as biennial FOBT recommendations do not apply to higher risk populations for whom more intensive methods of screening may be recommended. These participants receive a sealed envelope containing information about their survey results and are advised to discuss this with their primary care provider during their appointment.
3. Overdue for CRC screening: Average risk participants are asked to report whether they have ever had an FOBT or colonoscopy and, if so, when they had their most recent test. National Health and Medical Research Council guidelines recommend that average risk persons in the eligible age range undergo FOBT every 2 years [12]. Colonoscopy is not recommended as a routine screening test in Australia for those at average risk [12] but may be undertaken for other reasons (eg, the investigation of symptoms). Therefore, only those who report that they have not had an FOBT in the past 2 years or colonoscopy in the past 5 years are eligible for the trial.

The survey end screen contains a code that indicates to the research assistant if the participant is eligible for the trial. Eligible participants then receive the intervention if they attend the practice on an intervention day.

### Intervention

Immediately after completing the touchscreen survey, those participants identified as eligible for the trial and attending the practice on an intervention day are provided with a large envelope by the research assistant and advised to take it into their appointment with the primary care provider. This contains an FOBT kit accompanied by a referral form, instructions and a postage paid envelope addressed to a commercial pathology laboratory and a printed information sheet. The information sheet is a single-page A4 sheet using bold colored boxes to separate the information. The information encompasses topics including the type of screening test they should complete and how often they should complete this, what to do with the FOBT, what a positive FOBT result means, and credible websites where further information about bowel cancer screening can be obtained. The information sheet has a Grade 8 Flesch-Kincaid reading level.

When the participant takes the envelope into their appointment, the primary care provider explains the importance of FOBT and encourages the participant to complete the test.

### Usual Care

The usual care group receives no additional care. At the completion of the study, an information sheet similar to that provided to the intervention group is mailed to participants in the usual care group. This sheet contains additional information about how an FOBT can be sourced.

### Ethics and Dissemination

#### Data Management

Baseline data is collected using QuON open source survey software [37]. QuON is a software system specifically designed for the development of scientific surveys that allows data collection and aggregation of data via a Web browser. QuON survey data is instantaneously transmitted to the University of Newcastle secure server. No data is stored on the touchscreen device. Data is downloaded from QuON as a .csv file and imported directly to Stata IC 11.2 (StataCorp LLC) for statistical analysis. This form of data collection reduces the risk of data inaccuracy. Follow-up data is collected via computer-aided telephone interview using the QuON software system. This involves a structured interview of each participant guided by a preprogrammed electronic survey. The research assistant reads each question on the electronic survey to the participants and records all responses directly into the online interface. For most questions prespecified response options are provided to the participant (eg, yes, no, not sure).

#### Monitoring

Due to the size and duration of the study a formal monitoring committee and interim analysis is not required. The study is subject to the conditions of the University of Newcastle’s Human Research and Ethics Committee, including a random audit procedure to ensure the study is conducted in accordance with the approved ethics submission. This study has received ethical approval from the University of Newcastle Human Research and Ethics Committee (H-2014-0198) and has been registered with the Australian New Zealand Clinical Trials Registry (ANZCTR) [ACTRN12616001299493]. Any protocol amendments that may affect the conduct of the study, including changes of study objectives, study design, patient population, sample sizes, study procedures, or significant administrative aspects will require a formal amendment to the protocol. The modifications will be approved by the University of Newcastle Human Research Committee and updated as a new protocol version with the ANZCTR.

#### Confidentiality and Access to Data

Consent forms are stored in a locked filing cabinet at the University of Newcastle and accessible by one member of the research team. Data collected via touchscreen survey is instantly uploaded to a secure University of Newcastle server accessible only by a password-protected access system. Data will be retained for at least 7 years under these conditions at the University of Newcastle. FOBT results processed by the commercial pathology laboratory are electronically conveyed to the patient’s primary care provider by the password-protected online system. The pathology laboratory provides the researchers with the names of participants returning their FOBT but not individual test results. These details will be stored in a password-protected electronic file on the University of Newcastle server.

### Data Collection

#### Baseline Survey

For participants meeting the trial eligibility criteria, the following measures are collected in the touchscreen computer survey:

1. Demographic characteristics: age, gender, marital status, employment situation, highest education level, current private health insurance, current health care concession card holder.
2. Primary care provider visit characteristics: Participants are asked how many times they have seen their primary care provider in the past 12 months and whether they always see the same primary care provider, usually see the same primary care provider, or see whichever primary care provider is available.
3. Perception of personal risk of bowel cancer: Australian data indicates that 1 in 10 males and 1 in 15 females will develop CRC in their lifetime [3]. Participants are asked to select a response to the following statement: “I think my chance of being diagnosed with bowel cancer in my lifetime is...” Responses are 1 in 15, 1 in 25, 1 in 50, and 1 in 100.
4. Attitudes and intentions regarding CRC screening: Participants are asked to indicate their level of agreement with the following statements: (1) “Fecal occult blood testing is an effective way to detect bowel cancer,” (2) “I am confident I could complete an FOBT,” (3) “Most of my family aged 50 and older screen for bowel cancer,” and (4) “I intend to complete an FOBT in the next 2 years.” Response options are “strongly disagree,” “disagree,” “neither agree nor disagree,” “agree,” and “strongly agree.”
5. Knowledge of CRC screening recommendations: A 4-item study-specific instrument assesses knowledge of CRC screening recommendations using a multiple-choice format. The questions are prefaced by a description of average risk: “The following knowledge questions use the term ‘people at average risk of bowel cancer.’ Most people are at average risk of bowel cancer as they do not have a personal or strong family history of bowel cancer.” Each question has 4-6 response options. The questions were derived from National Health and Medical Research Council CRC screening guidelines [12]. The questions are (1) “At what age do you think people at average risk of bowel cancer should start screening?” (2) “What do you think is the recommended screening test for people at average risk of bowel cancer?” (3) “How often do you think a person at average risk of bowel cancer should have an FOBT?” and (4) “A positive FOBT means?” One point is awarded for each correct response.

#### Follow-Up Survey

Follow-up data is collected by telephone interview 6 weeks after study enrollment. This time point was selected based on data from the National Bowel Cancer Screening Program showing that participation rates begin to plateau within 6 weeks of invitations being sent [13].

CRC screening: Participants are asked to self-report whether they have completed any form of CRC screening (FOBT, colonoscopy, other). If the patient indicates they completed an FOBT, they are asked where they obtained this.

Knowledge of CRC: The 4-item instrument to assess CRC screening knowledge at baseline is also delivered at follow-up to detect changes in CRC knowledge.

Intervention group only: Acceptability of feedback sheet is assessed by the following questions: (1) “Did you read the feedback sheet?” (yes/no), if yes, (2) “Do you have any suggestions about how the feedback sheet could be improved?” (free response), (3) “Did you access any of the websites listed on the feedback sheet?” (yes/no), if yes, (4) “Which websites did you access?” and (5) “Do you think it would be helpful to receive information sheets from your primary care provider about other health issues?” (free response). Reasons for not being screened: Participants who report no screening are asked if there was a particular reason they did not use the kit provided at their primary care provider appointment (free response).

#### Process Measure

The researchers receive electronic notification of the names of participants returning an FOBT from the commercial pathology laboratory; however, no results are provided. This process measure will be used for an analysis of the sensitivity of self-reported screening.

#### Analysis and Sample Size

The age and sex of consenters and nonconsenters will be compared using the chi-square test for categorical variables and the *t* test or nonparametric equivalent for continuous variables. The proportion of participants completing CRC screening at the follow-up time point will be compared using a logistic regression model, including treatment group and site as independent variables. The correlation of observations induced by the design of the study will be accounted for through cluster robust variance estimation. A logistic regression will determine the characteristics associated with CRC screening. Differences in knowledge scores between the usual care group and the intervention group will be determined by ordinal logistic regression. For all tests, we will use 2-sided *P* values with a 5% significance level; exact *P* values will be reported. The primary analysis population will be all those who are randomized. Analysis will follow the intention-to-treat principle, with missing data imputed using multiple imputation. A sensitivity subanalysis of self-report versus pathology records in the intervention group will be conducted.

The sample size was calculated based on the primary aim. A sample size of 80 participants per arm will enable detection of a 25% increase in self-reported CRC screening for participants in the intervention group compared to 5% in the usual care group with 90% power at 5% significance. This calculation allows for a small design effect of 1.2 to allow for potential clustering by the design of the study (day of the week) and assumes on average 10 eligible participants will be available per day. Given that all participants eligible for randomization will have not participated in CRC screening via FOBT in the past 2 years or colonoscopy in the past 5 years, the underlying population prevalence of screening will not be considered in the sample size calculation.

## Results

At the time of submission, 5 primary care practices have consented to participate, with 100 participants enrolled in the study. Follow-up of participants has commenced, and it is anticipated all data collection will be complete by August 2017. Data analysis is in the preliminary stages. The authors will disseminate trial results through peer-reviewed publications and presentations at conferences.

## Discussion

### Strengths and Limitations

Previous research has demonstrated that multicomponent interventions are more likely to increase CRC screening participation than singular interventions [28]. Our study will test a multicomponent intervention using a gold standard RCT design across 5 primary care clinics. Very few intervention studies to increase colorectal cancer have been conducted in an Australian primary care setting.

## Abbreviations

ANZCTR: Australia New Zealand Clinical Trials Registry
CRC: colorectal cancer
FOBT: fecal occult blood test
RCT: randomized controlled trial
